# Radiological changes in STING‐associated vasculopathy of infancy treated with a JAK inhibitor

**DOI:** 10.1111/ped.70148

**Published:** 2025-08-28

**Authors:** Tomomi Sato, Yukihiro Nagatani, Yukiko Hirota, Tomoaki Kunitsu, Yoshihiro Maruo

**Affiliations:** ^1^ Clinical Education Center for Physicians Shiga University of Medical Science Otsu Shiga Japan; ^2^ Department of Pediatrics Shiga University of Medical Science Otsu Shiga Japan; ^3^ Department of Radiology Shiga University of Medical Science Otsu Shiga Japan; ^4^ Present address: Department of Pediatrics Maibara Community Medicine and Welfare Center Maibara Shiga Japan; ^5^ Present address: Department of Pediatrics Saiseikai Moriyama Municipal Hospital Moriyama Shiga Japan

**Keywords:** children, CT, interstitial lung disease, JAK inhibitor, SAVI

In 2014, STING‐associated vasculopathy with onset in infancy (SAVI) was first reported as a type I interferon dysregulation. SAVI is characterized by early‐onset systemic inflammation, cutaneous vasculopathy, and interstitial lung disease (ILD). The severity of ILD and the presence of recurrent lung infections determine the prognosis.^1^ Approximately 60 cases of this disease have been reported worldwide; there is also a case report in Japan.^2^, ^3^ Most patients with SAVI respond poorly to corticosteroids, immunosuppressive drugs, and biological agents. SAVI has a high mortality rate in the first 20 years of life, particularly in patients with severe lung involvement. However, promising results have been obtained with the use of Janus kinase (JAK) inhibitors.^2^, ^4^ Here, we discuss a 5‐year‐old Japanese patient with SAVI with respiratory failure and growth retardation associated with severe lung involvement who was treated successfully clinically and radiologically with the JAK1/2 inhibitor baricitinib.

A 5‐year‐old Japanese boy presented with a recurrent mild cough he had experienced since birth. He was of short stature and underweight. He had no abnormalities in his motor or intellectual development, but he soon became tired, fell asleep, and had difficulty climbing stairs from the first to the second floor. He had mild atopic dermatitis until the age of 2 years, after which he had no additional skin symptoms.

When the patient was 5 years old, a commissioned doctor at the nursery school noticed his severely low body weight (12.7 kg), retractive breathing, clubbed fingers, and suspected cardiovascular disease. That doctor referred him to our hospital. The patient's percutaneous oxygen saturation (SpO_2_) was 92%–95% at rest and upon waking and dropped to 80%–85% during exercise and sleep. Echocardiography showed mild pulmonary hypertension, but no signs of cardiac malformation or heart failure. Chest computed tomography (CT) showed extensive areas of trapped air in both the right and left lungs, faint ground‐light attenuation area in the right lower lobe, and a subpleural reticular lesion (Figure 1). Expectorants, bronchodilators, and macrolide antibiotics were administered and home oxygen therapy was prescribed. After the start of nasal dosing at 3 L/min, SpO_2_ was above 95% during normal exercise and 90% during exercise; however, hyperpnea and sinusoidal breathing remained. The lung CT findings led us to suspect SAVI, and a genetic diagnosis was made based on the V155M mutation in STING1. The patient's parents were informed about the effects and side effects of JAK inhibitors, and their written consent was obtained, and the informed assent was obtained from the patient. Baricitinib (4 mg/day), a JAK1/2 inhibitor, was orally administered as a treatment for SAVI. After starting baricitinib treatment, the patient was able to eat more food simultaneously, and his weight increased by 1 kg in 2 weeks. Depressed breathing gradually improved and disappeared within 2 months. Nasal oxygen was reduced to 1 L/min, he began to play more actively around with children of his own age, and he developed an increased desire to learn. KL‐6 was 5586 (U/mL), but after 6 months of treatment, it decreased to 3119 (U/mL), and there has been no change since. Six months later, chest CT showed that the entire air‐trapping area of the lung observed prior to treatment had reduced, and the faint ground‐glass attenuation area in the right lower lobe and the subpleural reticular lesion had almost disappeared (Figure 1). The patient's pulmonary symptoms improved clinically and radiologically with oral JAK inhibitors, and his quality of life improved markedly.

**FIGURE 1:**
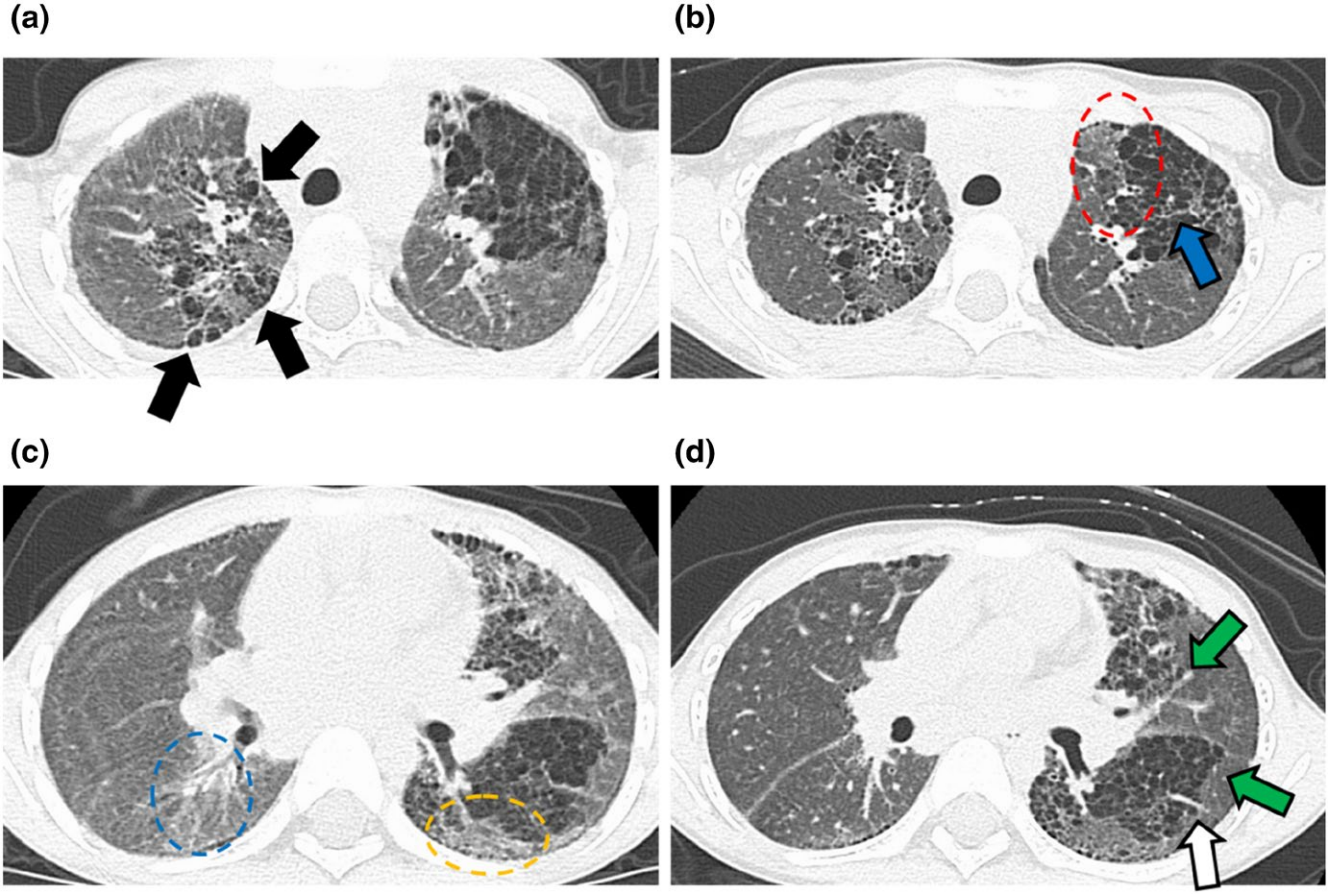
Tran‐axial thin‐slice CT images with 1‐mm thickness at the level of the aortic arch on the baseline (a) and followed up examination 6 month later (b), and the left atrium on the baseline (c) and followed up examinations (d) are demonstrated. In addition to the dorsal and mediastinal subpleural area of the right upper lobe (black arrows), air‐trapping area in the left upper segment seen on the baseline examination diminished mildly on the followed‐up examination especially for the inner area (red dotted circle). A pulmonary vessel in the left upper air‐trapping area got enlarged, and its border become distinct on the followed‐up examination (blue arrow). Compared with the baseline examination, mild decrease in the air‐trapping area in the left lingular and lower lobes was observed on the followed‐up examination (green arrows), with slight enlargement in pulmonary vessel inside the air‐trapping area in the left lower lobe (white arrows). These changes in image finding indicates the improvement in peripheral bronchial lesions and are very important. In addition, faint ground‐glass attenuation area in the right lower lobe (blue dotted circle) as well as subpleural reticular lesion (orange dotted circle) detected on the baseline examination almost disappeared on the followed up one.

Frémond et al. reviewed 61 SAVI cases. Complications included failure to thrive (75%, 41/55), recurrent fever (47%, 28/26), rash (69%, 40/58), ILD (79%, 46/58), and lung fibrosis (46%, 23/50). They analyzed the chest CT scans of 16 patients with SAVI and reported ground‐glass opacities (*n* = 13) with crazy‐paving patterns (*n* = 11) and cysts (*n* = 10). They also noted that many of the lesions were asymmetrical. In addition, many cases of pulmonary fibrosis occurred before adulthood. They also reported that all symptoms improved in the eight patients with SAVI who were followed over time, and that the best responses were seen in patients who were treated early, before irreversible lung damage occurred, confirming the importance of early diagnosis. Tolerance was good overall, but the risk of viral respiratory infections in patients with poor lung function needs special attention.^3^ Here we present a case of SAVI in a 5‐year‐old Japanese boy with no symptoms of skin or systemic inflammation. Although over 60 cases of SAVI have been reported since 2014, this is the second reported case in Japan.^2^, ^3^ Oxygen inhalation had a partial effect on our patient's respiratory symptoms. However, after starting oral baricitinib, he became more active, his respiratory status stabilized, and he gained weight. JAK inhibitors appeared to be effective even when imaging findings were poor. There have been a few reports of ILD in SAVI patients improving with JAK inhibitors, and in all the cases, treatment was started before the age of 10. It may be important to start treatment with JAK inhibitors in childhood.^5^, ^6^, ^7^

## AUTHOR CONTRIBUTIONS

Y.H. diagnosed and treated the patient; Y.N. was in charge of radiological findings and prepared the figure; T.S. discussed the case and wrote the manuscript; T.K. collected the data; and Y.M. supervised the project. All authors discussed the results and commented on the manuscript.

## CONFLICT OF INTEREST STATEMENT

The authors declare no conflict of interest.
